# Innovative Bitumen Modification Technology Using Industrial Waste Enamels in Asphalt Mixtures Production

**DOI:** 10.3390/ma19143054

**Published:** 2026-07-15

**Authors:** Miodrag Ristović, Jelena Gulicovski, Milan Kragović, Nenad Ristić, Ivica Ristović, Sanja Živković, Marija Stojmenović

**Affiliations:** 1 Institute of Nuclear Sciences “Vinča”, National Institute of the Republic of Serbia, University of Belgrade, 12-14 Mike Petrovića Alasa, 11351 Belgrade, Serbia; miodrag.ristovic@vinca.rs (M.R.); rocenj@vinca.rs (J.G.); m.kragovic@vin.bg.ac.rs (M.K.); sanjaz@vin.bg.ac.rs (S.Ž.); 2 Faculty of Civil Engineering and Architecture, University of Niš, Aleksandra Medvedeva 14, 18106 Niš, Serbia; nenad.ristic@gaf.ni.ac.rs; 3 Faculty of Mining and Geology, University of Belgrade, Đušina 7, 11000 Belgrade, Serbia; ivica.ristovic@rgf.bg.ac.rs

**Keywords:** asphalt mixtures, waste enamel, sustainable filler replacement, circular economy

## Abstract

This study presents, for the first time, an assessment of the dual role of waste enamels from heating device production in asphalt mixtures, as additives to modify euro bitumen (50/70) and as fillers, with a detailed analysis of their influence on properties of asphalt mixtures. Three types of enamels were investigated—premix (WEP), classic (WETM), and acid-resistant (WEART). Different characterization methods confirmed that these materials possess a borosilicate matrix enriched with various elements, including heavy metals (Cd, Cr, Cu, Ni, Pb, and Zn). Although classified as hazardous by-products, enamels replaced 100% of conventional stone dust filler, with confirmed leaching test. Their role in bitumen modification was interpreted through a structure–property approach: bitumen (4–6 wt.%) acts as a viscoelastic polymer-like matrix, while enamel particles serve as micro-scale reinforcements that govern binder–filler interactions. The results demonstrate that, despite their hazardous nature, waste enamels are compatible with asphalt technology containing 5 wt.% bitumen, achieving satisfactory stability, acceptable deformation response, and favorable volumetric characteristics. By valorizing industrial waste in this novel way, this study opens a sustainable pathway for transforming hazardous materials into functional components for the asphalt industry.

## 1. Introduction

The construction industry is one of the largest consumers of raw materials and energy worldwide, with road infrastructure playing a major role. Asphalt production alone consumes over 2 billion tons of aggregates and 102 million tons of bitumen annually, contributing significantly to natural resource depletion and greenhouse gas emissions [1,2,3,4]. The continuous depletion of natural aggregates and crude oil-derived bitumen not only threatens the long-term viability of traditional asphalt production but shows the urgency of developing sustainable alternatives and valorizing industrial residues [5,6,7].

On the other hand, engineering has been facing for years how to improve highway asphalt, including the mentioned savings in increasing asphalt life and performance. Standard paving bitumen in Europe are commonly classified according to penetration grades. most often used in the production of asphalt are 20/30, 35/50, 50/70, 70/100, and 160/220, depending on the intended pavement application [8]. However, to improve asphalt performance (resistance to deformation, elasticity, and durability), standard bitumen are often polymerized. A wide range of asphalts was made from modified polymer bitumen. The most famous polymer-modified bitumen (PMB) are bitumen with grades of PMB 25/55-55 or PMB 45/80-65 (the most commonly applied polymer is SBS—styrene-butadiene-styrene) [9,10,11]. In addition, some of the polymers added to bitumen as modifiers, including polyethylene, polypropylene, polystyrene, and polyvinyl chloride, are thermoplastic polymers. Polymer-modified bitumen improves its chemical and physical properties by adding polymer materials to the bitumen structure. This bitumen is used to increase the resistance, quality, and lifespan of asphalt layers in road construction, insulation, and sealing. Polymers that are added as additives to polymer-modified bitumen can improve bitumen properties, such as adhesion, softening point, and the brittleness of bitumen at low temperatures, among others. However, despite the high quality of the results in the use of these polymers, their relatively high price is a limiting factor.

Researchers have long investigated the application of industrial waste materials as alternatives to traditional mineral fillers and aggregates to address the environmental issues associated with the production of asphalt. In addition, scientists have been working for years to find an adequate source from waste by-products as filler that, in addition to the aforementioned environmental problems, such waste by-products will also enable good embedding in the bitumen structure in order to achieve satisfactory chemical and physical properties of asphalt. Fly ash, a by-product of the production of electrical energy in power plants, was first used as a mineral filler in hot-mix asphalt (HMA) mixtures in the United States in the early 1950s, which was the first instance of industrial waste being used as a filler replacement [12].

Building upon those early innovations [13,14,15,16,17], new research has increased the variety of industrial waste materials that can be used, such as copper tailings [18,19,20], ceramic dust [21,22,23], glass powder [24,25,26,27], and different metallurgical sludge [28,29,30,31]. It has been demonstrated that those materials improve the asphalt mixtures’ mechanical qualities, moisture resistance, and durability, while lessening the environmental cost of waste disposal and exploitation of primary resources.

On the other hand, although part of the environmental impact of asphalt production can be mitigated by replacing mineral fillers with industrial waste, the bitumen modification process that will replace polymerization with expensive additives is still a crucial component that significantly affects the durability, temperature susceptibility, and mechanical performance of asphalt mixtures. As a key component of asphalt, bitumen presents a complex blend of hydrocarbons and heteroatoms, primarily derived from crude oil. This is commonly described as a colloidal material, in which asphaltenes form a dispersed phase within a maltene matrix, giving rise to thermoplastic and viscoelastic behaviors [32]. Its polymer-like characteristics explain why modification with synthetic polymers, such as styrene-butadiene-styrene (SBS) and ethylene-vinyl acetate (EVA), has become standard for enhanced elasticity, stiffness, and durability [33,34,35]. However, as concerns about sustainability grow, scientists are looking more closely at substitute materials that resemble polymers, such as some powdered industrial wastes, which can offer comparable functional advantages without the environmental cost associated with producing virgin polymers [36,37]. The amorphous, glassy structures and chemical inertness of borosilicate-rich waste, like waste enamel, glass powder, and ceramic dust, can alter binder–filler interactions in ways like those of polymer additives [24,38,39]. It is known that the chemical reaction of bitumen and borosilicate in asphalt involves the modification of bitumen to enhance its performance [40]. Borosilicate additives can be incorporated into bitumen to improve its rheological properties, which are crucial for asphalt performance [41]. The interaction between bitumen and borosilicate can lead to the successful modification of bitumen, which leads to better durability and better performance of asphalt pavements, addressing issues like rutting and thermal cracking. These modifications are essential for developing more sustainable and environmentally friendly asphalt materials. By lowering the production costs related to primary materials and landfill disposal costs, the integration of industrial waste in the bitumen matrix not only lessens environmental degradation but provides financial benefits [42,43]. These methods, which support resource efficiency, waste reduction, and environmental sustainability in the infrastructure and construction industries, are also highly compatible with the principles of the circular economy. Nevertheless, despite their encouraging potential, there may be obstacles to the incorporation of industrial waste into asphalt, including variation in waste composition, possible environmental contamination, and regulatory restrictions that need to be properly managed [44,45,46].

Waste enamels, as borosilicate-rich by-products from heating device manufacturing, present a relatively novel and underexplored secondary raw material. The mentioned enamels share several traits with other glassy industrial residues that have been effectively repurposed in construction applications because of their high silica content and primarily amorphous structure [38,39,47]. In addition to aiding waste valorization, the incorporation into asphalt mixtures may promote a hybrid or polymer-like behavior in the bitumen matrix, creating new opportunities for asphalt systems modified with green polymers [38]. The modification process, including borosilicate, to enhance the engineering properties of bitumen, ultimately improves the overall performance of asphalt mixtures. The reuse of waste enamels has not attracted much scientific attention, especially when it comes to the process of the modification of bitumen and asphalt obtaining technology, despite its abundance and the pressure to reduce the landfill disposal of this hazardous industrial waste [48,49]. To the best of our knowledge, there are currently no studies investigating the use of waste enamels as sustainable additives in bitumen modification or their application as asphalt fillers.

Therefore, the purpose of this study is to provide the first systematic description of the dual role of waste enamels in asphalt mixture production—both as additives for the modification of 50/70 bitumen and as asphalt fillers—and to evaluate their effects on the mechanical, volumetric, and durability properties of the mixtures.

## 2. Materials and Methods

### 2.1. Materials

**Figure 1:**
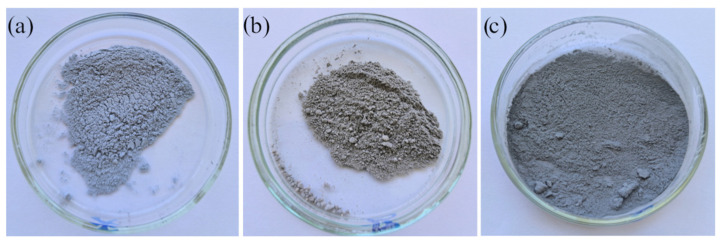
Waste enamels generated during the production process of heating devices: (**a**) premix technology (WEP), (**b**) classic technology (WETM), (**c**) acid-resistant technology (WEART).

The waste enamels used in this research were sampled from a heating device factory in the Republic of Serbia, with sampling conducted between October 2019 and January 2020. Since the aim was to use waste enamels in asphalt mixtures as additives and fillers, the preparation was focused on compatibility with standard asphalt practices, including drying, homogenizing, and sieving to obtain fine fractions, thereby ensuring consistent handling and mixture workability. The waste enamels were collected in their raw state from three different production processes of heating devices, then air-dried and homogenized to achieve uniformity and consistency. As a result, three initial enamel samples were generated during the production process of heating devices: premix technology (WEP), classic technology (WETM), and acid-resistant technology (WEART), as shown in Figure 1.

**Figure 2:**
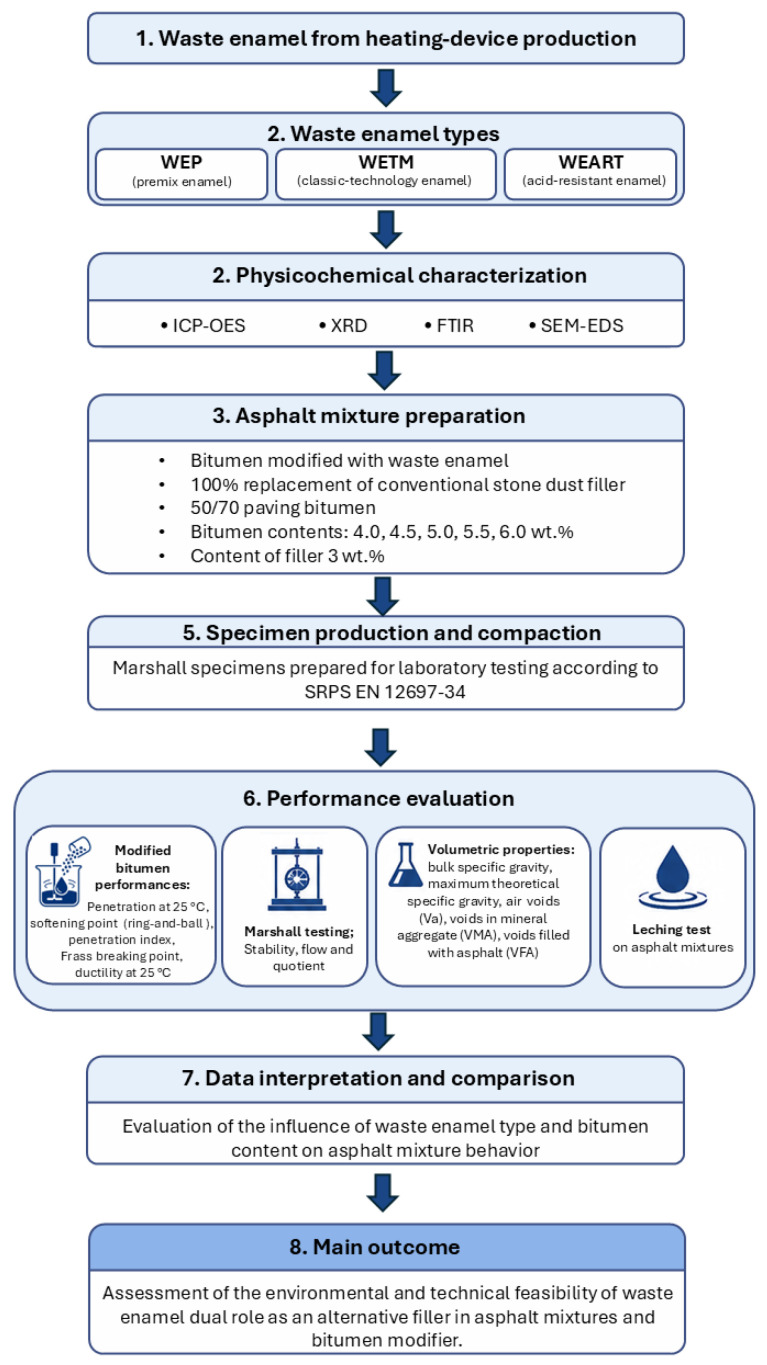
A schema of the experimental design and the main research steps of the present study.

Because waste enamels are materials with specific chemical compositions and characteristics different from conventional fillers (limestone or stone dust), their incorporation in the bitumen matrix may, to some extent, influence the properties of the asphalt mixtures. This is why we need a comprehensive characterization of the waste enamel and an evaluation of its interaction with the asphalt system. A schema of the experimental design and the main research steps is presented in Figure 2.

### 2.2. Physical–Chemical Analysis of Waste Enamels

#### 2.2.1. Chemical Analysis of Waste Enamels

Selected solid-state waste enamel samples were firstly solubilized via an appropriate chemical treatment according to the EPA 3051A method [50], for the rapid extraction of metals from the investigated samples. The elemental composition and heavy metal concentrations of both the untreated material and the leachate residues were then determined by using inductively coupled plasma optical emission spectrometry (ICP-OES) on an ICP spectrometer iCap 7400 duo, Thermo Fisher Scientific (Bremen, Germany).

#### 2.2.2. X-Ray Structural Analysis (XRPD)

Crystal phase composition was determined by X-ray powder diffraction (XRPD) using a Rigaku Ultima IV diffractometer (Rigaku, Tokyo, Japan) equipped with Cu Kα_1_ radiation (λ = 0.154178 nm) filtered through a graphite monochromator. Diffractograms were recorded over a *2θ* range of 5–90° in 0.02° steps at a scan speed of 5°/min. Crystalline phases were identified by processing the XRPD patterns in the DIFFRACplus software suite, where peaks, positions, and intensities were matched against the Powder Diffraction File (PDF) reference cards from the Joint Committee on Powder Diffraction Standards (JCPDS) of the International Centre for Diffraction Data (ICDD).

#### 2.2.3. Fourier-Transform Infrared Spectroscopy (FTIR)

Diffuse reflectance infrared Fourier transform (DRIFT) spectra were acquired at ambient temperature using a PerkinElmer Spectrum Quant spectrometer (PerkinElmer, Buckinghamshire, UK). Prior to analysis, each sample was mixed with anhydrous KBr in a 1:100 mass ratio to form a homogeneous pastille. Spectral data were collected over the 4000–400 cm^−1^ range, with appropriate resolution and baseline correction applied to identify characteristic vibrational bands corresponding to functional groups in the enamel’s matrix.

#### 2.2.4. Scanning Electron Microscopy–Energy Dispersive Spectroscopy (SEM-EDS)

A Carl Zeiss Jena JENAPOL-U polarizing microscope (Carl Zeiss Jena, Jena, Germany) operating in both transmitted and reflected light modes was used for the analyses. A scanning electron microscope (SEM; JSM-6610LV, JEOL Inc., Tokyo, Japan) running at an accelerating voltage of 20 kV was then used to examine the samples. Energy-dispersive X-ray spectrometry (EDS; Xplore 30, Oxford Instruments, Abingdon, UK) was used to support mineral identification. Based on internal standards, the detection limit was estimated to be around 0.1 weight percent. Potential compositional zoning was identified, and mineral homogeneity was evaluated using backscattered electron (BSE) imaging.

### 2.3. Characterization and Performance Testing of Asphalt Mixtures

The reference asphalt mixture AB 11 was produced using Euro bitumen for roads (grade 50/70), supplied by Gasprom Neft from the “Pančevo” Refinery, Pančeno, Serbia. The mineral composition included stone dust from the “Južna Morava” quarry in Dolac, Serbia, and crushed stone aggregates of three different fractions (0/4 mm, 4/8 mm, and 8/11 mm), all sourced from the “Budućnost” quarry in Preševo, Serbia. Euro bitumen 50/70 is a standard refined bitumen in the no polymerization additives basic form [51].

In order to obtain data on the potential possibility of bitumen modification using waste enamels, Euro bitumen 50/70 was modified with 3 wt.% WEP. The addition of WEP to the bitumen was used to assess whether the addition of fine enamel powder affects the basic consistency and temperature properties of the binder. The characterization of bitumen modified with waste enamel was conducted using the standards listed below. Penetration at 25 °C was performed according to SRPS EN 1426 [52], the softening point was determined by the ring-and-ball method according to SRPS EN 1427 [53], and the penetration index was calculated according to EN 12591 [54]. The Fraass breaking point was determined according to SRBS EN 12593 [55], and ductility at 25 °C was determined according to SRPS B.H8.615 [56]. All tests were conducted under standard laboratory conditions, and the obtained results were the specification limits for the 50/70 paving bitumen.

The proportion of the binder (bitumen) in this mixture varied between 4% and 6%, with increments of 0.5% to allow for performance comparisons. The composition of the reference material can be seen in Table 1.

In addition to the reference mixture AB 11, alternative asphalt mixtures incorporating industrial waste enamels (WEP, WETM, WEART) were produced. These mixtures used the same type of bitumen (Euro bitumen 50/70 from Gasprom Neft) and the same crushed stone aggregates from the “Budućnost” quarry. The only difference was the substitution of the traditional stone dust with three different types of enamel waste; the proportions can be seen in Table 2. The binder content for this mixture also varied from 4% to 6%, in steps of 0.5%, to match the testing conditions of the reference mixture.

Both the reference asphalt mixtures (AB 11) and the mixtures containing waste enamels as filler were created using the Marshall method according to the SRPS U.E4.014:1990 [57] to produce wearing layers from hot-process asphalt and SRPS EN 12697-34 standards [58]. The asphalt mixtures were made with binder (bitumen) contents varying incrementally from 4.0% to 6.0% by weight (at increments of 0.5%).

Mixtures were prepared under controlled laboratory conditions following standard protocols. Each mixture was created using the Marshall compaction method (50-blows per face), producing four samples per bitumen content level. After cooling to room temperature, these specimens underwent chemical, physical, and mechanical testing.

#### 2.3.1. Leaching Test

To evaluate the environmental behavior of the asphalt mixtures containing waste enamel, leaching tests were performed on selected asphalt mixtures. The leaching procedure was carried out in accordance with SRPS EN 12457-2 [59]. The leaching test was performed on crushed asphalt mixtures containing waste enamels at a liquid-to-solid ratio of 10 L/kg. The samples were then agitated for 24 h under laboratory conditions. The obtained eluates were filtered and analyzed by ICP-OES to determine the concentration of selected elements, including Al, B, Ba, Bi, Ca, Cd, Co, Cr, Cu, Fe, Mg, Mn, Ni, Pb, Sr, Zn, and Si. Determination of the concentration of the mentioned elements was repeated three times and the mean value is presented. This test was used as a way to assess the short-term release of heavy metals present in the original waste from the asphalt matrix under the applied laboratory conditions.

#### 2.3.2. Physical and Mechanical Testing

All procedures were carried out with calibrated equipment to ensure the accuracy and repeatability of the results. The test outcomes were statistically analyzed, and the results for the asphalt mixtures containing waste enamel fillers were compared against the standard reference mixture (stone dust filler). Table 3 shows the tests that were conducted to evaluate the mechanical and volumetric properties of the asphalt mixtures.

The asphalt mixtures’ mechanical performance under traffic load was tested using Marshall Stability (S), as described in standard SRPS EN 12697-34 [58]. The test is performed on a standardized cylinder specimen that has been submerged in a water bath or air oven that has a constant temperature of 60 °C for 30 to 40 min. Then, it is placed into the Marshall loading frame that applies load at a constant deformation rate of 50.8 mm/min where the maximal load shows the Marshall Stability (kN) and the deformation at failure shows the Marshall Flow (mm).

Tangential flow (F_t_) was determined according to the same standard as an additional deformation parameter derived from the Marshall load-deformation curve. It represents the nominal deformation obtained by extrapolating the initial tangent of the curve forward to the stability load and back to zero load. The value expresses the mixture of elastic and early viscoelastic deformation that occurs before the onset of failure.

During the Marshall test, total flow (F_T_) was simultaneously recorded as the total deformation of the specimen from the beginning of loading to the point of maximum load at 60 °C. This parameter represents the total strain that the mixture experiences during loading, including plastic, viscoelastic, and elastic strain.

The Marshall Quotient (MQ) represents the flexibility index of a mixture, expressed as a ratio of Marshall Stability to Marshall Flow, which is represented in Equation (1):

(1) $$MQ=\frac{Marshall\;Stability\;(kN)}{Marshall\;Flow\;(mm)}$$

#### 2.3.3. Bulk Specific Gravity and Volumetric Characteristics

One of the first tests that is needed to determine the characteristics of asphalt mixtures is the determination of the bulk specific gravity (G_mb_) of an asphalt mixture, as it is used for the calculation of important volumetric parameters: air void content (V_a_), void in material aggregate (VMA), and void filled with asphalt (VFA).

According to standard SRPS EN 12697-6:2020 procedure B [60], this test is performed as follows:

(a) Determination of the mass of the dry specimen (m_1_);
(b) Determination of the density of the water at test temperature to the nearest 0.0001 mg/m^3^ (r_w_);
(c) Immersion of the sample in the water-bath at the known test temperature, and allowing the full saturation of sample surfaces (usually 30 min, and maximum 3 h);
(d) Determination of the mass of the saturated sample when it is immersed (m_2_);
(e) Removal of the sample from water, drying it from water drops that have adhered;
(f) Determination of the mass of the saturated surface wiped specimen in an air immediately after drying (m_3_);
(g) Calculation of the bulk specific gravity (G_mb_) according to Equation (2):

(2) $$G_{mb}=\frac{m_{1}}{\left(m_{3}-m_{2}\right)}\times r_{w}$$

#### 2.3.4. Maximum Theoretical Specific Gravity

The theoretical maximum specific gravity (G_mm_), also known as the maximum specific density, represents the specific gravity of an asphalt mixture when all air voids are eliminated. This value is important as it calculates the void content in compacted asphalt, and it is used to evaluate the degree of compaction. In this paper, the theoretical maximum density of all asphalt mixtures was determined following the standard set by SRPS EN 12697-5:2019, Procedure A [61].

The procedure uses a vacuum pycnometer. A representative dry sample of asphalt mixture (around 100 g) is weighed and put in a calibrated pycnometer flask, after that the flask is filled with water at 25 °C until the whole sample is submerged. A vacuum of at least 4 kPa is applied for approximately 15 min so it can remove all the trapped air from the sample. The theoretical maximum specific gravity is then calculated by dividing the mass of the dry sample by the volume of displaced water, which is shown in Equation (3):

(3) $$G_{mm}=\frac{m_{\mathrm{dry}}}{V_{\mathrm{pyc}}}$$

#### 2.3.5. Void Content in Compacted Mixtures

Void content (V_a_), or air voids, in asphalt mixtures refers to the percentage of the total volume that is not occupied by aggregates or binder.

The void content was determined in accordance with SRPS EN 12697-8 [62] by comparing the bulk density of the asphalt mixture with the theoretical maximum density of the same mixture, as shown in Equation (4):

(4) $$V_{a}(\%)=\left(\frac{G_{mm}-G_{mb}}{G_{mm}}\right)\times 100$$

#### 2.3.6. Voids in Mineral Aggregates

A critical parameter in asphalt mixtures is the number of voids in mineral aggregates (VMA), which represents the volume of void spaces between the aggregates in the asphalt mixture, and it is shown as a percentage of the total volume of the mixture. The VMA was calculated using the bulk density of the compacted asphalt mixture sample and the known bulk specific gravity of the aggregate blend as it is shown in Equation (5):

(5) $$VMA(\%)=100-\left(\frac{G_{mb}\times P_{s}}{G_{agg}}\right)$$

The percentage of aggregate (G_agg_) in the asphalt mixture is the mass fraction of the mineral aggregates relative to the total mass of the mixture, including the bitumen binder, and P_s_ is the percent of the total aggregate in the asphalt mixture.

#### 2.3.7. Voids Filled with Asphalt

The percentage of void space in the mineral aggregate that is filled with the asphalt binder in an asphalt mixture is known as the voids filled with asphalt (VFA). The VFA is directly connected to the air void (V*_a_*) and the void in the mineral aggregates shown in Equation (6): as the air decreases, the VFA increases as the air pockets that were primarily in the mineral skeletal structure are filled with the binder. This can be expressed as follows:

(6) $$VFA(\%)=\left(\frac{VMA-V_{a}}{VMA}\right)\times 100$$

## 3. Results and Discussion

The interaction between bitumen and filler, such as stone flour or waste enamels containing heavy metals, is key to the formation of bituminous mastic, which binds the larger aggregate in the asphalt mixtures. Bitumen in this process has a role not only as a binder, but as a matrix for the permanent “trapping” of hazardous substances such as heavy metals. The quality of this connection depends on the physical and chemical properties of the waste enamels, which is why a detailed characterization of all applied industrial waste enamels is presented below.

### 3.1. Results of the Physical–Chemical Analysis of the Waste Enamels

#### 3.1.1. Chemical Analysis of the Waste Enamels

The results of the chemical analysis (Table 4 and Table 5) revealed that all three waste enamel samples contained elevated concentrations of several heavy metals (Cd, Cr, Cu, Ni, Pb, and Zn), in excess of the threshold values for non-hazardous classification according to Serbia’s “Rulebook on the categories, testing and classification of waste” (Official Gazette of the Republic of Serbia, Nos. 56/2010, 93/2019, 39/2021, and 65/2024) [63]. The obtained results confirm the classification of enamel waste as a hazardous material, requiring specialized disposal or immobilization procedures under current legislation.

As mentioned, determining the concentration of heavy metals and their corresponding oxides in borosilicate waste, as well as their interaction with bitumen, is crucial in the formation of asphalt mixtures, particularly during the solidification and stabilization processes [64]. In this interaction, bitumen not only facilitates the physical binding of waste enamel particles but serves as a matrix for the permanent immobilization of heavy metals. The immobilization mechanism is based on the fact that bitumen is highly hydrophobic, which is essential for waste containing heavy metals. From the perspective of a physical barrier, bitumen envelops borosilicate particles with an opaque layer, preventing water from contacting the heavy metals and leaching them into the environment. From the perspective of chemical inertness, borosilicate is inherently stable, and bitumen does not react aggressively with metals, thereby preventing the formation of new, soluble compounds.

Further, when it comes to the impact of heavy metals on bitumen, the presence of heavy metals and their oxides in borosilicate waste can act as a catalyst in the bitumen matrix [65,66]. Certain metals (such as copper or iron) can accelerate the oxidation of bitumen, and therefore its accelerated aging, making it more brittle over time. However, metal oxides in the waste enamels can lead to a change in the softening point, often acting as hardeners, and increasing the viscosity and resistance of the mastic (mixture of bitumen and waste enamels as fillers) to high temperatures.

Interaction with heavy metal-contaminated borosilicate can also alter the behavior of asphalt in terms of rheological properties and stability. Heavy metals may sometimes enhance the adhesion between borosilicate particles and bitumen, acting as filler “bridges,” although this effect depends on the specific concentration of metal ions. Furthermore, bitumen is considered to be one of the most effective matrices for long-term stabilization, as it does not crack under frost conditions like cement matrices, thereby reducing the risk of subsequent metal release.

Thus, with the ecological aspect, the main goal of the interaction between the bitumen and the particles of the waste enamels is the leaching tests. Under laboratory conditions, bitumen indicates the possibility of immobilizing metals such as lead, cadmium, and chromium from borosilicate waste, thereby converting hazardous waste into usable construction material. The limit values for heavy metals in bitumen are not defined as a fixed percentage that the bitumen “receives”, but through the immobilization capacity, that is, the ability of the bitumen matrix to prevent their leaching into the environment. In practice, this is checked by leaching tests according to the SRPS EN 12457-2 standard [59]. When borosilicate waste is trapped in bitumen, the final product (asphalt mixture) must release metals at concentrations lower than those prescribed for non-hazardous waste with strict observance of limit values of leaching (solidification). According to new EU waste management regulations that apply starting from the year 2025 [67], any material containing heavy metals used in construction must have a waste characterization report confirming that the level of leaching is below the remedial values for land. Additionally, according to the latest guidelines for the circular economy, materials obtained from this interaction (bitumen + hazardous waste) receive the label “End-of-Waste” [68] only when the report confirms that the leaching values are at the level of natural aggregate.

Finally, as part of this research, a high concentration of oxides, like SiO_2_ and B_2_O_3_, dominates the matrix (Table 4 and Table 5), giving the waste enamels a chemically inert structure, ensuring that heavy metals are evenly distributed and that their activity is controlled, regardless of the classification. Their low solubility and strong bonding in a silicate network greatly reduce the leaching potential of most elements when encapsulated in an asphalt mixture, which is indicative of its chemical stability. When the metals Al, Ba, Ca, Cd, Cr, Cu, Fe, Mg, Mn, Ni, Pb, Zn, and their oxides are found in the borosilicate matrix, together with bitumen, they act as modifiers and catalytic centers. Al, Ca, and Mg form stable oxides/hydroxides in the matrix, act as fillers, and improve the mechanical resistance of bitumen [69]. Fe, Mn, Ni, Cu, and Cr, as transition metals, can catalyze the oxidation of bitumen, accelerating the decomposition of aromatic and aliphatic components [69]. Zn and Ba are often used as stabilizers and, in the matrix, can contribute to resistance to UV and thermal aging [70]. Pb and Cd are chemically active and considered problematic due to toxicity; however, in the borosilicate matrix, they prevent metal migration and ensure uniform distribution in the bitumen [70], and the negative ecological effects are limited. Immobilization of the mentioned elements within the asphalt binder matrix can successfully lower environmental risks while concurrently enhancing mechanical performance, which is comparable with earlier research application of vitreous industrial waste (such as glass powder and ceramic dust) [21,44,71].

In summary, the combination of the noted metals and their oxides in a borosilicate matrix with bitumen can supports its compatibility with asphalt mixtures as an alternative filler, but the control of metals must be careful due to environmental constraints. The leaching results (Table 7) indicated that the asphalt matrix can physically encapsulate waste enamel particles and support their immobilization in a stable asphalt mixture that immobilizes heavy metals within the asphalt matrix. Therefore, even though borosilicate waste enamels are officially categorized as hazardous waste, their chemical composition and leaching results indicate that they are highly compatible with asphalt technology, as presented in Section 3.2.

#### 3.1.2. X-Ray Structural Analysis (XRPD)

**Figure 3:**
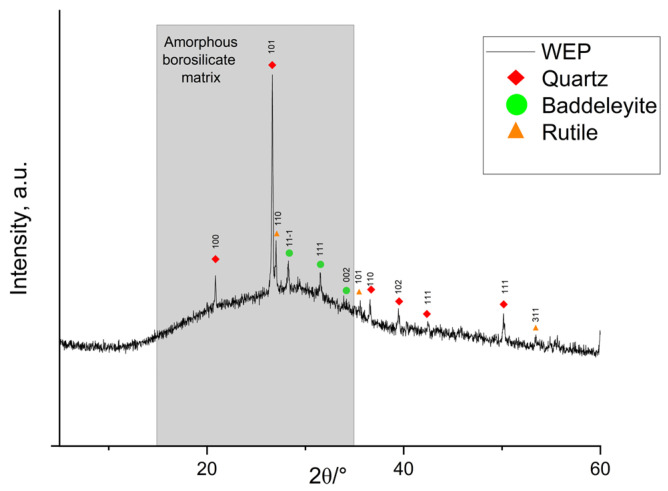
XRD diffractograms of waste enamel, the WEP sample.

**Figure 4:**
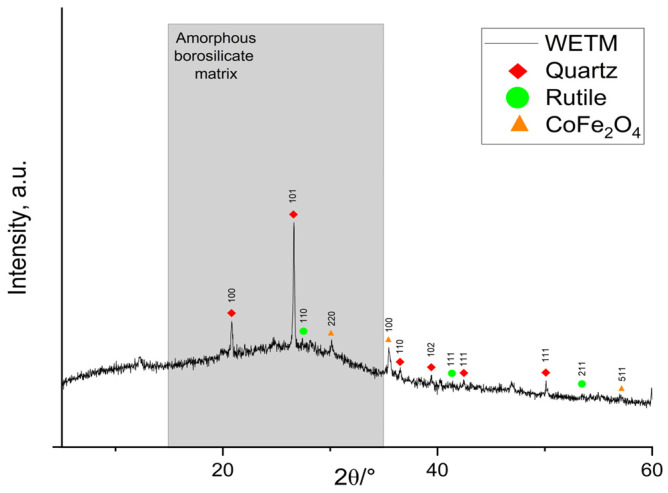
XRD diffractograms of waste enamel, the WETM sample.

**Figure 5:**
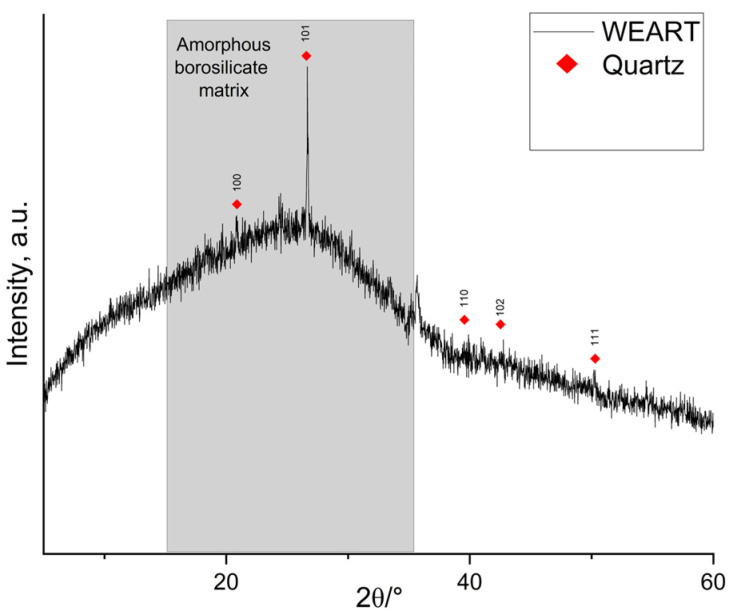
XRD diffractograms of waste enamel, the WEART sample.

The results of the XRD analysis of the samples WEP, WETM, and WEART are presented in Figure 3, Figure 4 and Figure 5. All three samples exhibit a dominant amorphous broad peak between roughly 15° and 35° *2θ*, which is typical of a borosilicate matrix.

As seen in Figure 3, the WEP sample exhibits noticeable quartz (SiO_2_, ICSD No. 39830) peaks, with the strongest reflection occurring at about 26.6° *2θ*. Furthermore, baddeleyite (ZrO_2_, ICSD No. 60900) and rutile (TiO_2_, ICSD No. 33846) forms are found, indicating that zirconium and titanium compounds were added to improve opacity and chemical durability during the production of heating devices.

Regarding the asphalt mixtures, ZrO_2_ and TiO_2_ in the borosilicate matrix do not react directly with bitumen in the sense of a classical chemical reaction, but are distributed in it as nanocomposite additives, which improve the mechanical, thermal, and chemical properties of the composite [72,73,74]. When ZrO_2_ and TiO_2_ are found together in a borosilicate matrix with bitumen, their behavior represents a combination of stabilizing and catalytic effects [74]. Their role is more physical–chemical, leading to structure stabilization, and increase corrosion resistance and photocatalytic activity [73]. In particular, ZrO_2_ remains stable, does not react directly with bitumen, and its role is to increase the mechanical strength, wear resistance, and thermal stability of the composite [74]. TiO_2_ under UV light can catalyze the oxidation of the organic components of bitumen, which means that exposure to sunlight can accelerate the aging of bitumen but, at the same time, it contributes to the degradation of surface pollutants [72]. The synergy of ZrO_2_ and TiO_2_ in the borosilicate matrix improves the adhesion between the bitumen and the mineral matrix, making the composite more resistant to moisture and mechanical stress. Together in a borosilicate matrix, these two oxides create a mechanically and chemically very resistant composite, but careful balance is required to avoid accelerated aging of the bitumen.

The presence of quartz is also confirmed by Figure 4, which depicts WETM. However, it also shows peaks for rutile (TiO_2_) and for CoFe_2_O_4_ (ICSD No. 29630), a cobalt ferrite spinel phase. Such a composition indicates the application of additives based on titanium and cobalt, probably for functional improvement and coloring of heating devices. While the rutile reflects crystallized TiO_2_ under oxidizing conditions, the CoFeO_4_, which peaks at about 35.5° *2θ*, indicates a high-temperature solid-state reaction between iron and cobalt oxides during the production of heating devices.

The presence of TiO_2_ and CoFe_2_O_4_ in the borosilicate incorporated in bitumen of asphalt mixtures can significantly improve pavement performance, as TiO_2_ increases resistance to aging and UV degradation [75,76], while CoFe_2_O_4_ contributes to mechanical strength and potentially enables the photocatalytic degradation of pollutants from the road surface [77]. Studies have also shown that asphalt containing TiO_2_, in addition to better resistance to moisture and aging, also has a higher softening point, which extends the life of the pavement [75,76]. In this combination, borosilicate can serve as a carrier and stabilizer for the mentioned particles, preventing their agglomeration and enabling an even distribution of catalytic centers in the bitumen. Everything mentioned ensures the long-term stability and considerable savings, because the application of expensive nanoparticles significantly increases the costs of asphalt production. Thus, the application of waste enamels containing a borosilicate matrix with the presence of TiO_2_ and CoFe_2_O_4_ particles in asphalt is a promising technology which potentially combines improved mechanical performance with photocatalytic properties. In practice, this would mean longer-lasting, more resilient, and more environmentally friendly roads, especially in urban areas where the pollution and load on the roadways are the greatest.

While WEART, shown in Figure 5, lacks the definition of peaks, the ferrite and rutile phases seen in WETM, it displays a similar glassy background with crystalline peaks for quartz and a strong peak at 35.6° *2θ*, suggesting the presence of minor oxidized or spinel-type phases due to Fe–Cr–Cu enrichment, which is consistent with the ICP and EDS analysis. When a Fe–Cr–Cu alloy or mixture is found inside the borosilicate matrix added to bitumen, its influence is primarily physical–mechanical, with a specific contribution to thermal stability. These metals act as high-density rigid fillers. Inside the glass matrix, they form a composite “core” that increases the stiffness modulus of the bitumen [78,79]. In addition, Cu has exceptional thermal conductivity and its presence helps the bitumen to distribute heat more quickly and evenly [78,79], while Cr inside the alloy provides passivation, making the metal particles resistant to oxidation even in acidic environments [78,79]. Although copper and chromium ions are potentially toxic, in the borosilicate matrix, they are “trapped” and can meet “End-of-Waste” status. Therefore, the presence of Fe–Cr–Cu in the borosilicate matrix can transform the waste into a high-performance reinforcer that improves the bearing capacity and thermal regulation of asphalt, without the risk of chemical contamination.

#### 3.1.3. Fourier-Transform Infrared Spectroscopy (FTIR)

**Figure 6:**
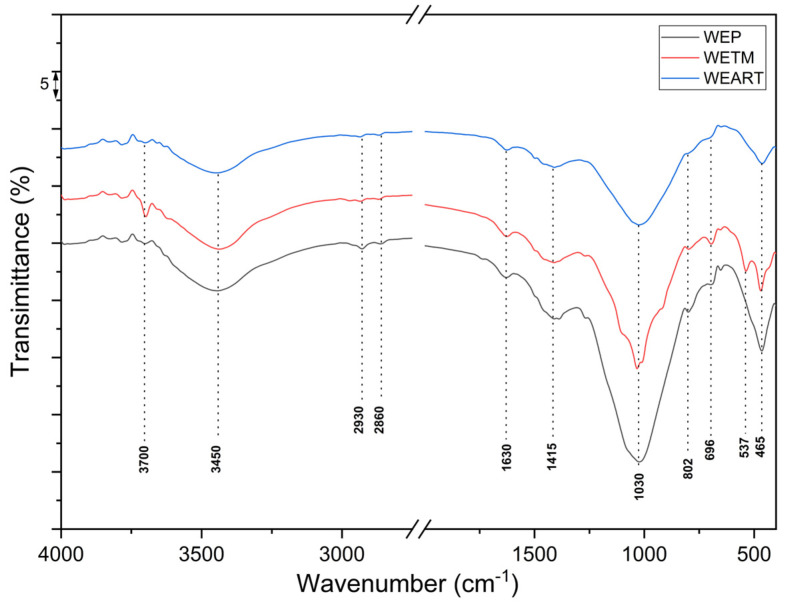
FTIR spectra of the waste enamels WEP, WETM, and WEART samples.

The structural characteristics of the waste enamel samples (WEP, WETM, and WEART) were examined by Fourier-transform infrared spectroscopy. Spectra were acquired in the 4000–400 cm^−1^ range, and the representative spectra are shown in Figure 6. Based on the obtained results, there are no notable differences in the overall spectral profiles of the examined samples, suggesting a broadly similar vitreous structure with only minor compositional variance.

For all investigated samples, the following spectral bands were recorded: 465, 696, 802, 1030, 1415, 1630, 2860, 2930, 3450, 3700 cm^−1^; an additional spectral band was recorded for the WETM sample at 537 cm^−1^. Broad bands located at 3400–3500 cm^−1^, together with the weak features near ~3700 cm^−1^, are attributed to O–H stretching vibrations associated with surface hydroxyl groups and adsorbed water [80]. Molecular water is furthermore proven by the presence of a band observed at 1630 cm^−1^, which shows O–H–O bending vibrations [81].

The most intense band, located at 1030 cm^−1^ in all three samples, is assigned to the asymmetric stretching vibration of Si–O–Si bonds [82]. Additionally, bands were detected at 802 cm^−1^ that represented symmetrical Si–O–Si stretching, and the bands located at 465 cm^−1^ (Si–O–Si bend) confirm the predominance of a glassy matrix [83], which is consistent with the amorphic character identified by XRD. Low intensity features near 1415 and 696 cm^−1^ were observed in all the samples and are classified as borate-related vibrations (BO_3_ structural units) [84]. Even though the high concentration of boron is present in all three samples, indicated by the ICP results, the borate and borosilicate (Si–O–B) contributions are not visible, as they are known to overlap with the silicate (Si–O) vibrations, especially in the 1000–1100 cm^−1^ region, and therefore cannot be fully resolved from the dominant SiO_2_-related bands [84,85].

Weak absorption bands detected at 2930 and 2860 cm^−1^ were assigned to the C–H stretching vibrations of alkanes, indicating the presence of organic residues [86]. The bands observed near 537 cm^−1^ correspond to metal–oxide vibrations superimposed on the borosilicate matrix. When comparing the heavy metal concentrations across all three samples, it can be noted that the WETM sample exhibits slightly higher concentrations than the WEP and WEART samples, which may explain the appearance of the peak near 537 cm^−1^, characteristic of metal–oxide vibrations. However, due to the complex composition of the enamel material used in the heating industry, these spectral features could not be attributed to specific metal oxides.

#### 3.1.4. Results of Scanning Electron Microscopy–Energy Dispersive Spectroscopy

**Figure 7:**
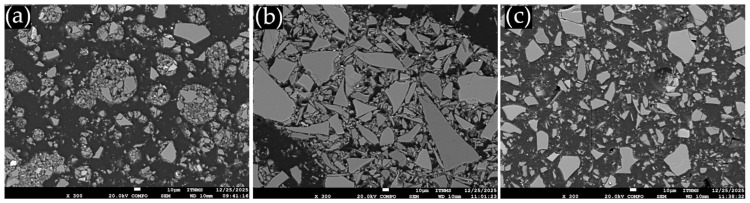
SEM micrographs of the waste enamels: (**a**) WEP, (**b**) WETM, and (**c**) WEART samples.

Representative SEM micrographs of the waste enamels (WEP, WETM, WEART) are shown in Figure 7. All of the samples consist mostly of angular, polygonal shards with sharp edges. However, the dispersion of grains is different between the samples.

From the SEM image of the sample WEP presented at Figure 7a, it can be seen that there is a highest degree of agglomeration, with numerous rounded clusters composed of tightly packed tiny fragments surrounding large particles. On the other hand, in the SEM image of the sample WETM (Figure 7b), a higher fraction of large flake-shaped shards and a locally dense particle packing are visible, indicating a broader particle size distribution and frequent contact between grains. The SEM image of the sample WEART shown in Figure 7c exhibits a comparatively more homogeneous distribution of particles, with fewer compact agglomerates.

**Figure 8:**
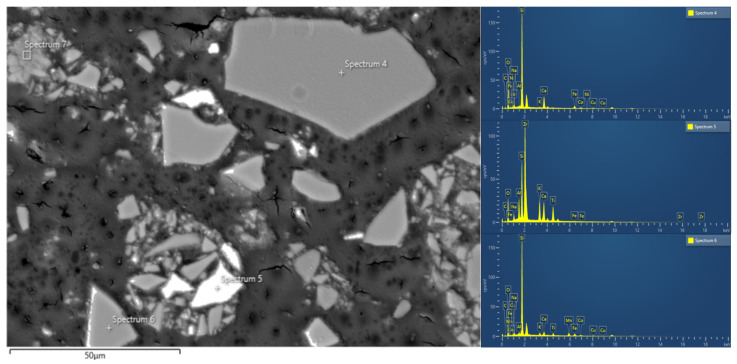
SEM micrographs with EDS spectra of the WEP sample.

**Figure 9:**
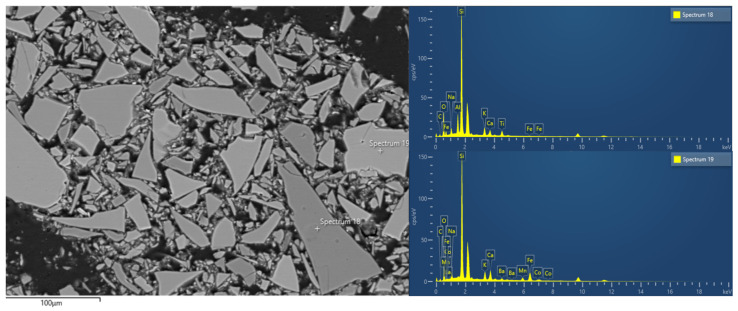
SEM micrographs with EDS spectra of the WETM sample.

**Figure 10:**
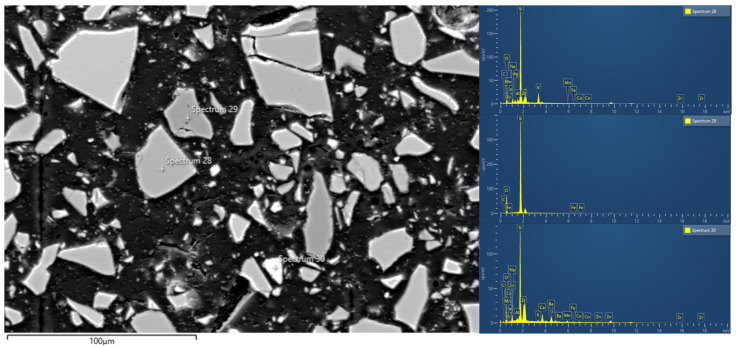
SEM micrographs with EDS spectra of the WEART sample.

The EDS analysis confirms that the dominant phase in all samples is a Si–O-rich borosilicate matrix (Figure 8, Figure 9 and Figure 10) containing variable amounts of network modifiers (Na, K, Ca) and metals (such as Ti, Zr, Fe, Cu, etc.). Although the ICP analysis indicates a high boron concentration, no boron signals were detected by EDS. This is expected because boron is a light element whose characteristic X-ray emission is at very low energy and, as a result, it is often below the practical detection limits of conventional SEM-EDS systems [87,88]. Carbon detected in some spectra is attributed primarily to the mounting medium.

The results of the EDS point analysis of the sample WEP is present at Figure 8 where the dominant presence of a silicate-based matrix modified with alkali and alkaline elements is visible. EDS spectra 4 and 6 (Figure 8) show high content of O (≈44.7–47.9 wt.%) and Si (≈26.2–29.2 wt.%), with small amounts of Na, Ca, and Al which are below 5 wt.%, accompanied by transition metals, such as Fe around 3 wt.%, Mn around 3.4 wt.%, Co around 1 wt.%, and Cu between 0.6. and 1.2 wt.%. In addition to the standard borosilicate matrix, a distinct inclusion was identified (Figure 8, Spectrum 5), characterized by the strong enrichment of Zr (27 wt.%) and K (5.6 wt.%). This is consistent with the Zr-bearing phase embedded within the surrounding borosilicate matrix, which aligns with the XRD analysis.

In the sample WETM, shown in Figure 9, the results also indicate a dominant silicate-based glassy matrix. Spectra 18 and 19 show high content of Si (≈30 wt.%) and O (≈34.3–42.8 wt.%). Additionally, the spectra show a small amount of Na, K, and Ca that hover around 3 wt.%. Spectrum 18 indicates the presence of Ti (2.9 wt.%), Al (4.5 wt.%), and a small amount of Fe (0.16 wt.%). While Spectrum 19 in Figure 9 shows a much higher content of Fe (8.8 wt.%), and small amounts of Co (1.01 wt.%), Ba (2.32 wt.%), and Mn (2.5 wt.%).

An additional EDS analysis was performed (Figure S1) to establish the chemical composition of the inclusion found in the glass matrix. The most distinct inclusion (Spectrim 21) is Ti-rich (≈60 wt.%), with a measurable V at around 4 wt.% and Sb close to 2 wt.%. This is consistent with the XRD analysis of the WETM sample, in which the crystalline phases of TiO_2_ (Rutile) were detected. Cd-bearing grains were clearly identified, indicating cadmium-based colorant residues in the enamel waste. Spectrum 22 contains Cd at approximately 29 wt.%, whereas Spectrum 23 shows even stronger Cd enrichment around 47 wt.%, confirming that Cd occurs primarily as discrete pigment grains. The obtained results were further supported by the ICP analysis, which indicates an elevated Cd concentration in the WETM sample.

Similar to the WEP and WETM samples, the results of the EDS analysis of the sample WEART shown in Figure 10 (Spectra 28, 29, and 30) shows a high amounts of Si (≈24.8–41.1 wt.%) and O (38.2–48.1 wt.%), as well as a spectrum of Fe with a low intensity of 0.12 wt.% that is visible (Spectrum 29). Spectrum 28 is accompanied by a low amounts of K (6.31 wt.%), Zr (3.12 wt.%), Na (2.62 wt.%), Mg (1.16 wt.%), and Al (1.10 wt.%), while Mn (0.11 wt.%), Fe (0.26 wt.%), and Co (0.17 wt.%) are also visible. By contrast, Spectrum 30 showed high amounts of Si and O and elevated amounts of Zr (10.34 wt.%), as well as Ba, Ca, Na, Ti, Mn, Co, Zn, Fe, K, and Al, with weight percentages of 4.25, 3.47, 3.23, 2.09, 1.24, 0.85, 0.77, 0.49, 0.43, and 0.58 wt.%, respectively.

An additional EDS analysis was performed on the WEART sample (Figure S2) to identify inclusions. Spectra 31, 32, and 33 show pronounced enrichment in transition metals, especially Fe, Cu, and Cr. In the first analyzing site (Spectrum 31), the analyzed area contains nearly equal amounts of Cr and Cu, 14 wt.% and 13 wt.%, respectively, along with a slightly higher Fe concentration of 18 wt.%. At the second analyzing site (Spectrum 32), a notably higher Fe content was observed (28 wt.%), while Cr is present only at low levels (6 wt.%). High carbon content (41 wt.%) is attributed to the mounting medium, since the inclusions are small and the EDS includes the surrounding background material. Similar to Spectrum 31, Spectrum 33 shows nearly identical Cr and Cu content (both around 9 wt.%), along with a higher Fe content of approximately 13 wt.%, and in low amounts Mn and Si (≈1.5 wt.%).

Thus, the defined compositions are consistent with localized metal-rich inclusions, most likely originating from pigment residue or processing-related metal–oxide-bearing particles, embedded within the enamel matrix. All results obtained by the SEM-EDS analysis are in accordance with the results obtained by the XRD, FTIR, and ICP analyses, which gave us clear guidelines to approach the examination of the application of waste enamels based on borosilicate as additives and filler substitutes in the production of asphalt mixtures. Detailed tests of the asphalt mixture parameters are given in the next sections.

### 3.2. Characterization and Performance of the Asphalt Mixtures

#### 3.2.1. Properties of Bitumen Modified with Waste Enamel WEP

The properties of the Euro bitumen 50/70 modified with the WEP enamel are presented in Table 6. The addition of the waste enamel to the bitumen had a slight stiffening effect as the penetration was reduced from 64 to 60 mm. At the same time, the softening point increased, suggesting an improved consistency at elevated temperatures. This is also evident when we are looking at the penetration index, indicating a lower high-temperature susceptibility of the modified binder. The Fraass breaking point changed from −12 °C to −10 °C, showing a small reduction in low temperature flexibility; however, the value remains within the required criteria. Under laboratory testing conditions, the ductility remained above 100 cm.

Similar stiffening trends have been reported for advanced asphalt binder modifiers, such as carbon nanotubes, where their addition has been shown to decrease penetration and to increase the softening point of bitumen. With the addition of nanotubes, Haq et al. [89] noted a reduction in ductility, as well as the wider application in asphalt pavements is limited by relatively high material cost. On the other hand, the addition of the WEP enamel produced a moderate stiffening effect, suggesting that the WEP enamel may provide a binder stiffening effect while avoiding some of the drawbacks reported for certain high-cost modifiers. Nevertheless, this comparison should be considered preliminary, and additional rheological, aging, and long-term binder performances will be evaluated.

#### 3.2.2. Leaching Test Results

After leaching of the asphalt mixtures, even though the original waste enamel samples contained elevated concentrations of several heavy metals, the results obtained for the eluates showed that Cd, Co, Cr, Cu, Ni, and Pb were below the detection limits in all analyzed samples. Zn was detected only in the WETM mixture at a low concentration of 0.17 mg/kg while it remained below the detection limit in the other mixtures. The aforementioned behavior of asphalt mixtures is supported by the result of the leaching test shown in Table 7.

#### 3.2.3. Performance of the Asphalt Mixtures

One of the main knowledge gaps in the asphalt industry is the understanding of the relationship between the chemistry, rheology, morphology, and microstructure of bitumen and different additives and fillers. It is often stated that this interaction is key to a complete understanding of asphalt mixture design and engineering performance. Researchers have been trying to solve the mentioned problem through the application of different methodologies in order to improve the characteristics of bitumen, the incorporation of various additives and fillers, and therefore, asphalt properties.

In order to better understand the possible interactions between bitumen and additives and fillers in the asphalt mixture, several facts are presented. Namely, in the colloids science, it is initially hypothesized that, in bitumen structure, by hierarchy, it contains a core, micelles, and aggregates consisting of asphaltenes [90]. This theory classifying the structure of bitumen based on the propensity of asphaltenes to form nanoaggregates and clusters [91,92], known as the Yen–Mullins model, was partially validated [93,94]. The mentioned model helps to understand why bitumen has a colloidal nature and why additives and fillers can change its properties, affecting the stability and size of these aggregates. Asphaltenes (the most polar and heaviest molecules in bitumen) are key in forming the structure of bitumen and its interaction with fillers and additives. The right combination of additives and fillers can control the behavior of asphaltenes, resulting in asphalt mixtures with better moisture resistance, cracking, and aging. So, the initial idea is that asphaltenes, as the most polar and heaviest fraction of bitumen, tend to aggregate. According to the Yen–Mullins model, which is one of the most important concepts in the science of colloids applicable to bitumen, nuclei are formed from asphaltenes. Then micelles are formed in the form of asphaltenes as the nuclei are surrounded by resin layers that stabilize them and prevent their uncontrolled aggregation. The next stage is represented by aggregates/clusters that are formed by connecting multiple micelles into larger structures; and finally, the appearance of anoaggregates, which indicate the tendency of asphaltenes to form larger aggregates, which affects the rheological properties of bitumen. The colloidal matrix is thus formed, where all these structures are dispersed in maltenes (aromatic and saturated hydrocarbons) form a “solution” in which micelles float. Specifically, in the asphalt mixture, asphaltenes in combination with heavy metals in a borosilicate matrix create complex interactions that affect chemical stability, mechanical resistance, and durability of pavement structures. As has already been demonstrated in practice [95,96,97], by milling the particles down to nanometer dimensions, a significant strengthening of the material can be achieved; the use of nanoparticle reinforcements is widespread. In this case, the nanoparticles of heavy metals can stabilize asphaltene micelles and improve aging resistance, where the most important effects are the binding of metals to aromatic and heteroatomic groups of asphaltenes, adsorption on the surface of the borosilicate matrix, and potential catalytic oxidation. Thus, this model allows us to explain why bitumen is not a simple liquid but a colloidal system and helps in the development of additives that can stabilize or modify asphalt mixtures. Additionally, this model provides a starting point for research into bitumen aging and its interaction with fillers and polymers. However, the eclectic nature of bitumen, a complex and diverse structure that shows a combination of different physical and chemical properties, depending on its composition and environmental conditions, makes it difficult to comprehend and engineer but, at the same time, forms a unique viscoelastic, adhesive, long-lasting, and adaptable construction binder [98,99].

Thus, the effect of waste enamels on the asphalt mixtures’ performance can be interpreted using a structure–property approach in which the bitumen (asphalt binder) is treated as a polymer-like viscoelastic matrix, while the waste enamel (filler) acts as a micro-scale reinforcing phase that controls the behavior of the binder–filler mastication. In this scope, the key question is how the waste enamels’ chemistry, structure, and morphology (identified by ICP-OES, XRD, FTIR, and SEM-EDS) translate into the properties of asphalt mixtures, which are reflected in volumetric and mechanical responses. The outcome of all the performance testing that was performed on the asphalt mixtures is shown in Table 8.

##### Marshall Stability

**Figure 11:**
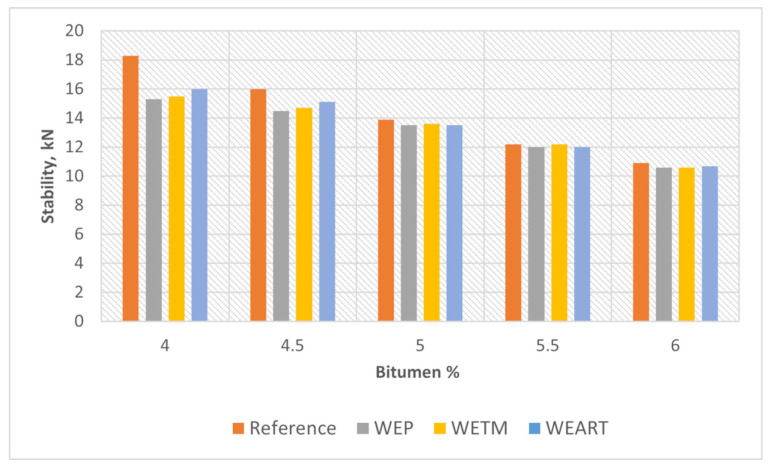
Results of the Marshal Stability Test.

The Marshall Stability test demonstrated that the complete replacement of conventional stone dust filler with enamel waste does not compromise the structural performance of the asphalt mixture. As shown in Figure 11, all test samples exceeded the minimum stability requirement of 8 kN, as specified in SRPS U.E4.014:1990 [57] and other international standards. Figure 11 shows that waste enamel does not increase the stability of the asphalt mixture, but it does not degrade it under the international requirements. As shown in Figure 11, the largest difference was observed for the WEP mixture at 4.0 wt.% bitumen, where stability was approximately 16% lower than that of the reference mixture. With an increase in bitumen content, the difference in stability of the asphalt mixture with stone powder and the asphalt mixtures with waste enamel is decreasing. At 5.5% bitumen, the stability values of the waste enamel mixtures were very close to those of the reference mixture, with WETM showing the same Marshall stability value as the reference. Even though the waste enamel did not improve the Marshall stability, especially at lower binder content, the mixtures still complied with the required stability criterion.

##### Marshals Flow Test

**Figure 12:**
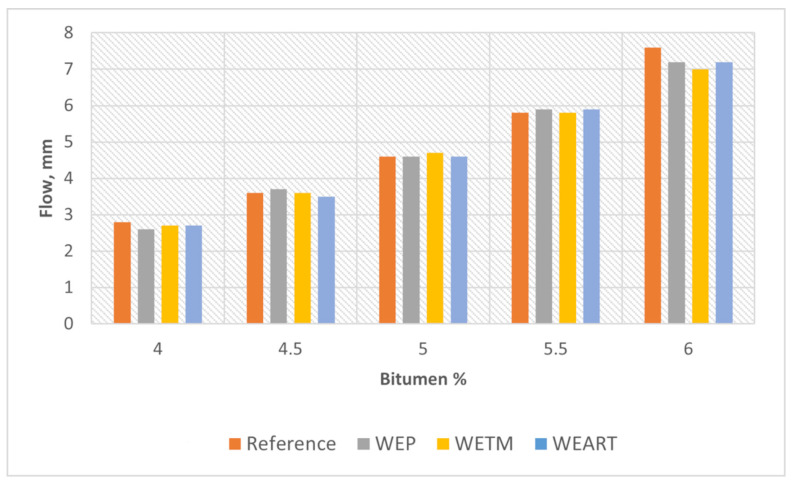
Results of the Marshal Flow Test.

Flow test (F) is a complementary test to the Marshall Stability, as it is defined as a test of the plastic deformation (in millimeters) that happens when the asphalt sample receives the maximal load. It is a good indicator of asphalt’s flexibility and its ability to accept thermal expansion, concentration, and minor structural movement without cracking. It can also give information about the binder content in the mixture. A high flow value can indicate an overly rich mixture with too much binder, which can lead to bleeding and rutting of the asphalt. On the other hand, a low flow value indicates a stiff, brittle mixture prone to cracking and poor fatigue resistance. It is typically recommended that the Marshall flow values fall within the range of 2.0 and 4.0 mm. As expected, the flow values shown in Figure 12 increase with the rise of bitumen content, which indicates greater plastic deformation under load. At bitumen concentrations of 4.0% to 5.0%, all mixtures generally fall within or just slightly above the acceptable flow range. On the other hand, at 5.5% and especially 6.0%, all mixtures exceed the 4.0 mm upper threshold, with the maximum values of over 7 mm. From this, it can be seen that the higher content of bituminous binder gives the mixture more plastic properties.

Asphalt samples with the waste WEP and WEART exhibit flow values identical to the reference mixture at 5.0% bitumen, and the asphalt mixture with WEART matches the reference mixture when the concentration of bitumen is 5.5%. The biggest deviation in the flow value is at 6.0% bitumen, even though the corresponding differences in the Marshall Stability were less pronounced than those observed at 4.0%.

##### Void Content in Compacted Mixtures

Void content is a critical parameter in evaluating the quality, durability, and performance of an asphalt mixture. The presence of the air void in the material allows for thermal expansion and contributes to the mixture’s resistance to deformation. Excessive void content can lead to moisture infiltration, oxidation, and premature deterioration, while the absence of air voids can result in bleeding and a reduction of skid resistance. The optimal air void content is typically between 3% and 6% for a medium grade asphalt mixture.

**Figure 13:**
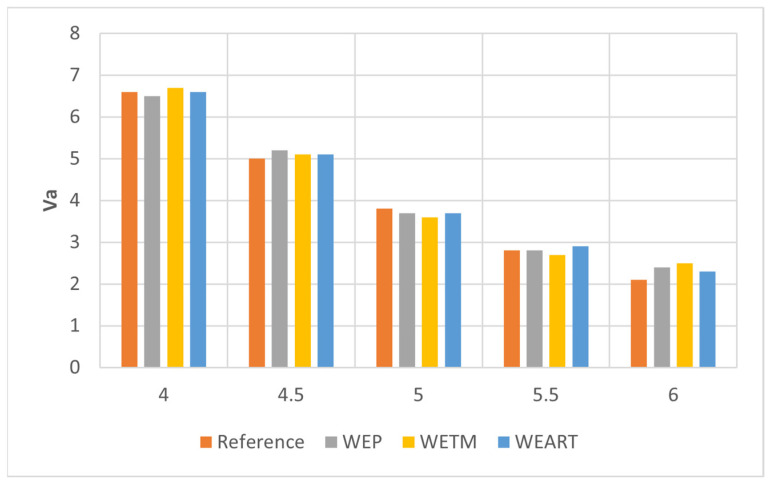
Void content of asphalt mixtures.

The air void content (V_a_) of asphalt mixtures prepared with varying bitumen contents (4–6%) for the reference mixture and those containing the waste enamel fillers (WEP, WETM, WEART) is shown in Figure 13. For every mixture, there is a general downwards trend in V_a_ as the binder content rises, suggesting better compaction and aggregate interlock as more bitumen fills the void spaces. The behavior of the waste-modified variants and the reference mixture is comparable, indicating that the addition of enamel waste does not substantially affect the mixture’s compatibility.

All mixtures show higher void ratios (6.5–7%) at 4% binder content, indicating that there is not enough binder to fully coat the aggregate surface. This is outside of the ideal void content range described by SRPS EN 13108-1:2017 [100]; the optimal range between 3% and 6% is between 4.5% and 5.5% binder. This ensures sufficient air volume for thermal expansion and durability without sacrificing resistance to deformation. At 5.5% of binder, the V_a_ falls below 3%, which could suggest possible bleeding hazards and decreased skid resistance in high-temperature conditions.

##### Voids in the Mineral Aggregate (VMA)

**Figure 14:**
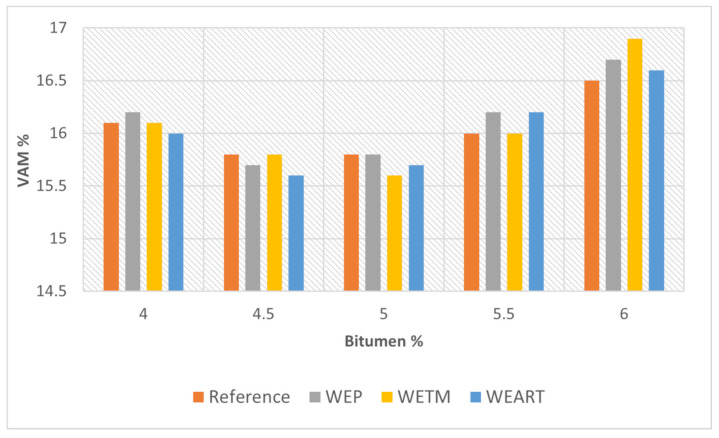
Voids in the Mineral Aggregate.

The VMA determines the spaces available to accommodate the binder; this directly affects the mixture’s resistance to moisture damage, durability, and flexibility. If there is too small VMA, the asphalt mixture will not be able to accept enough binder and will not be able to provide long-term cohesion, which will lead to early aging, cracking, or raveling. The VMA is represented in Figure 14 and shows the percent of voids in the mineral aggregate that range between approximately 15.5% and 17%, which satisfies the minimal/optimal range for asphalt mixtures, which is 15% [101]. Among the tested mixtures, WETM at 6.0% bitumen showed the highest VMA value, suggesting that this formulation creates a more open aggregate structure capable of accepting a greater volume of binder.

The slight dip in the VMA, observed between 4.5% and 5.0% of bitumen, could be explained by compaction dynamics and binder lubrication effects. At a lower 4.0% bitumen content, there is not enough binder to lubricate and identify the aggregate structure. This then makes the compaction less effective, leaving more voids between particles. Air has a hard time escaping, so it leads to high voids in the mineral aggregate [102].

##### Voids Filled with Asphalt (VFA)

From a performance standpoint, a higher VFA improves durability by reducing permeability and enhancing cohesion between aggregate particles. It indicates that the binder adequately coats and binds the mineral aggregate, which minimizes the possibility of disintegration, stripping, or premature cracking. An appropriate value of the VFA is important as it helps the mixture to resist stress, such as rutting, fatigue cracking, and moisture damage, especially in regions where there is a big difference in temperature and in locations with high traffic volume. On the other hand, an excessively high VFA may indicate excessive binder relative to the void space, increasing the risk of deformation, especially under high loads or in warmer climates.

**Figure 15:**
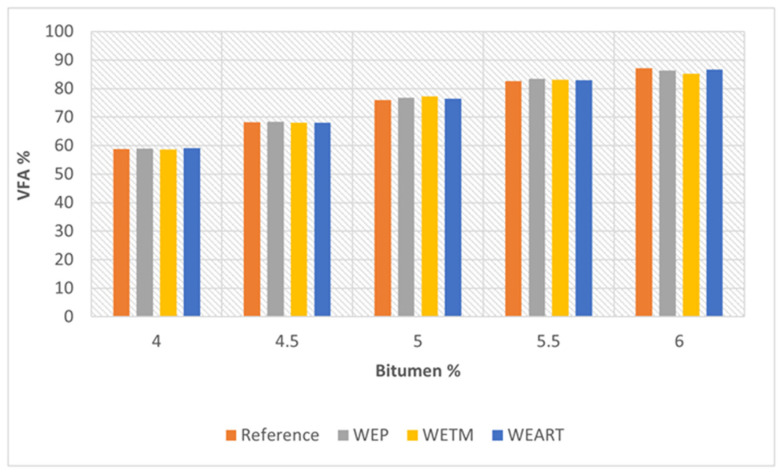
Voids Filled with Asphalt.

The percentage of voids in the mineral aggregate filled with asphalt binder (VFA) is shown in Figure 15. As expected, the results show a clear increase in the VFA for all mixtures as the bitumen content rises. The VFA values range from 58% to 60% at 4.0% binder, which is indicative of relatively low binder saturation, is common in drier mixes with more air voids. The VFA has a dramatic rise of about 20% as the binder content increases to 5.0%, which indicates a notable improvement in binder distribution and coverage. At 6.0%, the VFA value surpasses 85%, which is close to full saturation of the voids in the material. Guided by the SRPS EN 13108-1 [100], where it is suggested that the minimal VFA value should be between 70% and 86%, it can be said that any asphalt mixture with the bitumen content higher than 5% satisfies this condition.

### 3.3. Influence of Waste Enamels Properties on the Performance of the Asphalt Mixtures

The XRD, ICP, FTIR, and EDS results confirm that the borosilicate matrix with localized, compositionally distinct inclusions, supporting the concept that metal-bearing components occur primarily as discrete particles embedded within an overall phase.

At the mixture scale, these characteristics mainly influence performance through physical packing and mastic formation, rather than through any nanoscale reinforcement of chemical compatibility. This is evidenced by the volumetric trends: air voids decrease systematically with increasing binder content, and the optimal V_a_ (3–6%) is achieved in the mid-binder interval (4.5–5.0%), whereas at 4% binder the voids are high (6.5–7%), and at 5.5%, V_a_ drops below 3%, indicating an overly rich condition with a risk of bleeding and rutting. These volumetric shifts represent a bridge between microstructure and mechanics, as they reflect how effectively the binder coats the aggregates and how the mastic fills the internal voids.

The mechanical results follow the same logic of structure and properties. All mixtures exceed the minimum Marshall stability requirement (8 kN), demonstrating that the structural integrity is not affected by the complete replacement of stone dust with enamel filler. The differences between the enamel-filled mixtures and the reference mixture decrease with increasing binder content, reaching essentially identical stability at 5.5% binder; the largest deviation occurs at 4% binder (especially for WEP), which is consistent with insufficient binder availability and less early mastic development. The Marshall thickness increases with the binder content and exceeds the commonly recommended upper limit of 5.5–6.0% (with maximum > 7 mm), confirming that excessive binder shifts the release response of the compositions to a harmless form. Overall, the combined evidence supports that waste enamels act as a functional microfiller of an internal viscoelastic polymer-like matrix: when the volume shows adequate mastic formation (≈4.5–5.5%), the mixture exhibits good bonding properties and benchmark performances, while the low and high binder extremes are primarily determined by mastic deficiency or binder supersaturation.

## 4. Conclusions

In this study, for the first time, the double role of waste enamels from the manufacture of heating devices in the production of asphalt mixtures was proven. Waste enamels in their raw state, which were sourced from three heating device production technologies (WEP, WETM, and WEART), were successfully applied as additives for modifying euro bitumen (grade 50/70) and as asphalt fillers. By the ICP-OES, XRD, FTIR, and SEM-EDS methods, it was proven that waste enamels show a borosilicate matrix with the presence of various elements, including heavy metals (Cd, Cr, Cu, Ni, Pb, and Zn). The asphalt mixtures were prepared in accordance with SRPS EN standards, and they used Euro Bitumen 50/70 in binder contents ranging from 4.0% to 6.0% by weight. In accordance with the obtained results, we can conclude the following:

1. Although classified as a hazardous by-product, the waste enamel WEP was successfully applied as additives for modifying euro bitumen (grade 50/70) replacing expensive commercial additives. Moderately improved bitumen consistency at elevated temperatures while maintaining standard specification compliance, with a slight low temperature trade-off, was confirmed.
2. Although classified as a hazardous by-products, waste enamels have, at the same time, very successfully replaced 100% of conventional stone dust filler without significant degradation of the mechanical and volumetric properties of the asphalt mixtures. The short-term leaching test indicates a good immobilization of hazardous elements in laboratory conditions.
3. The bitumen modification process, by incorporating waste enamels as additives, was successfully interpreted using a structure–property approach in which the bitumen (asphalt binder) is treated as a polymer-like viscoelastic matrix, while the waste enamel with heavy metals (filler) acts as a micro-scale reinforcing phase that controls the behavior of the binder–filler mastication.
4. Marshall stability and flow, bulk and maximum specific gravity, air voids (V_a_), voids in mineral aggregates (VMA), and voids filled with asphalt (VFA) were used to evaluate the mechanical and volumetric properties. The findings demonstrated that all enamel-modified mixtures outperformed the 8 kN minimum Marshall stability requirement, with performances identical to the reference mixture at 5.5% binder content. At 5.5% bitumen, the classical technology (WETM) mixture performed similarly to the reference, indicating the best possible binder–filler interaction. By contrast, mixtures containing 6.0% binder showed higher flow values, which may indicate plastic deformation. Thus, there was a risk of plastic deformation at high binder levels, as evidenced by the flow values rising with binder content and surpassing the upper 4 mm threshold at 6.0%.
5. Volumetric analyses showed that, while the VFA increased steadily with bitumen content reaching 85–87% at 6.0%, the VMA initially decreased because of improved aggregate compaction. Thus, an adequate VMA (15–17%) and a favorable VFA (>70% at ≥5% binder) were confirmed by volumetric analysis. These results demonstrate that using waste enamel does not alter volumetric equilibrium and validate successful void filling. Like other glass–ceramic residues, the amorphous structure helps to immobilize potentially hazardous components within the asphalt matrix.
6. Finally, the best overall performance was achieved at 5% binder content, where the mixtures showed optimal stability, favorable volumetric balance, and the most efficient binder–filler interaction.

All things considered, even though waste enamels were characterized as hazardous waste, the achieved results indicated that enamels can be considered as potential alternative additives and fillers in asphalt mixtures. The parameters indicate that waste enamels are highly compatible with asphalt technology, with careful control of heavy metals due to environmental constraints. The present study demonstrated a satisfactory Marshall and volumetric behavior, together with the low release of heavy metals under the laboratory leaching conditions as a primary feasibility assessment. Nevertheless, applied waste enamels present a potential practical and sustainable substitute for additives and mineral fillers, supporting waste valorization and the ideas of the circular economy, including multi-objective optimization of their application in the asphalt industry. By valorizing industrial waste in this novel way, this study opens a sustainable pathway for transforming hazardous materials into functional components of the asphalt industry. Further research should include the determination of optimum bitumen content with the influence of waste enamel content as additives and filler, durability, aging assessment, rutting resistance, and long-term leaching tests under simulated field conditions.

## Figures and Tables

**Table 1:** Composition of aggregate in reference mixture AB 11.

Name of Basic Materials	Mixture Proportion
Stone dust	3%
0/4 mm aggregate	54%
4/8 mm aggregate	24%
8/11 mm aggregate	19%
Total:	100%

**Table 2:** Composition of aggregate in asphalt mixture with waste enamels.

Name of Basic Materials	Mixture Proportion
Waste enamels (WEP, WETM, WEART)	3%
0/4 mm aggregate	54%
4/8 mm aggregate	24%
8/11 mm aggregate	19%
Total:	100%

**Table 3:** Mechanical and volumetric tests performed on the asphalt mixture samples.

Tests	Standards
Marshall Stability at 60 °C	SRPS EN 12697-34:2013 [58]
Flow tests at 60 °C	
Marshall quotient determination	
Tangential and total flow at 60 °C	
Bulk specific gravity	SRPS EN 12697-6:2020, procedure B [60]
Maximum theoretical specific gravity of asphalt mixtures	SRPS EN 12697-5:2019, procedure A [61]
Void content in compacted mixtures	SRPS EN 12697-8:2019 [62]
Voids in mineral aggregates	
Voids filled with asphalt	

**Table 4:** Chemical analysis of the waste enamel samples (mg/kg).

Parameter	WEP	WETM	WEART	Reference Values [63]
Al	6494 ± 27	27,391 ± 173	5053 ± 33	/*/**
B	47,300 ± 740	48,786 ± 220	47,043 ± 350	/*/**
Ba	1071 ± 6	interference	2248 ± 16	100/* 300/**
Bi	<0.06	<0.06	<0.06	/*/**
Ca	24,020 ± 540	28,600 ± 440	17,770 ± 290	/*/**
Cd	<0.06	1526 ± 8.6	<0.06	1/* 5/**
Co	6260 ± 100	2511.8 ± 9.7	6081 ± 10	/*/**
Cr	46.7 ± 1	860 ± 47	1572 ± 34	10/* 70/**
Cu	4910 ± 120	814 ± 13	4440 ± 45	50/* 100/**
Fe	20,986 ± 58	18,883 ± 47	31,469.0 ± 45	/*/**
Mg	2090 ± 58	2640 ± 3	1636 ± 5	/*/**
Mn	23,760 ± 170	10,020 ± 40	29,500 ± 210	/*/**
Ni	290.4 ± 0.1	419.1 ± 4.6	318.5 ± 2.8	10/* 40/**
Pb	56.4 ± 0.8	51.8 ± 0.6	38.8 ± 6.2	10/* 50/**
Sr	126.4 ± 4.6	74.9 ± 0.8	300.8 ± 3.8	/*/**
Zn	185 ± 21	5557 ± 13	1104 ± 7	50/* 200/**
Si	43,330 ± 250	53,488 ± 270	53,380 ± 280	/*/**

According to “Rulebook on the categories, testing and classification of waste (Sl. glasnik 56/2010, 93/2019, 39/2021)”: * values valid for the disposal of non-reactive hazardous waste at non-hazardous landfills in cassettes, not intended for disposing of biodegradable waste; ** values valid for the disposal at a hazardous waste landfill.

**Table 5:** The content of oxides in the waste enamel samples (mg/kg).

Parameter	WEP	WETM	WEART
B_2_O_3_	152,310.7	157,095.8	151,483.2
Fe_2_O_3_	30,003.68	26,997.03	44,991.23
Al_2_O_3_	12,270.41	51,755.29	9547.644
SiO_2_	92,695.87	11,440.98	114,195.8
Na_2_O	239,975	59,950	<0.06
K_2_O	68,185	41,242.5	6081
CaO	33,608.78	40,017.12	24,863.78
MgO	3465.638	4377.648	2712.815
SO_3_	2992.5	572.5	318.5
P_2_O_5_	55,175	5182.5	53,380

**Table 6:** Properties of the Euro bitumen 50/70 no polymerization additives basic form and Euro bitumen 50/70 bitumen modified with the WEP enamel.

Test	Criteria	Euro Bitumen 50/70	Euro Bitumen 50/70 with WEP	Standard
Penetration at 25 °C, mm	50–70	64	60	SRPS EN 1426 [52]
Softening point °C	46–54	51.5	53.5	SRPS EN 1427 [53]
Index of penetration	−1.5 do +0.7	−0.23	−0.1	SRPS EN 12,591 [54]
Fraass breaking point, °C	≤−8	−12	−10	SRPS EN 12,593 [55]
Ductility at 25 °C, cm	≥100	>100	>100	SRPS B.H8.615 [56]

**Table 7:** Leaching results of the asphalt mixtures containing waste enamel (mg/kg).

Elements	WEP	WETM	WEART	Reference Values [63]
Al	6.44	6.92	6.97	/*/**
B	56.73	61.41	57.56	/*/**
Ba	3.49	2.61	3.29	100/* 300/**
Bi	<0.003	<0.003	<0.003	/*/**
Ca	187.3	171.5	158.15	/*/**
Cd	<0.001	<0.001	<0.001	1/* 5/**
Co	<0.001	<0.001	<0.001	/*/**
Cr	<0.003	<0.003	<0.003	10/* 70/**
Cu	<0.001	<0.001	<0.001	50/* 100/**
Fe	0.22	0.29	0.21	/*/**
Mg	42.56	39.88	40.6	/*/**
Mn	0.17	0.14	0.17	/*/**
Ni	<0.003	<0.003	<0.003	10/* 40/**
Pb	<0.003	<0.003	<0.003	10/* 50/**
Sr	0.87	0.82	0.83	/*/**
Zn	0.17	<0.002	<0.002	50/* 200/**

According to “Rulebook on the categories, testing and classification of waste (Sl. glasnik 56/2010, 93/2019, 39/2021)”: * values valid for the disposal of non-reactive hazardous waste at non-hazardous landfills in cassettes, not intended for disposing of biodegradable waste; ** values valid for the disposal at a hazardous waste landfill.

**Table 8:** Results of Performance Tests on the Asphalt Mixtures.

Mixture Type	Reference Asphalt Mixture					WEP					WETM					WEART				
**Bit (%)**	4.0	4.5	5.0	5.5	6.0	4	4.5	5	5.5	6	4.0	4.5	5.0	5.5	6.0	4	4.5	5	5.5	6
**S (kN)**	18.3	16.0	13.9	12.2	10.9	15.3	14.5	13.5	12	10.6	15.5	14.7	13.6	12.2	10.6	16	15.1	13.5	12	10.7
**F (mm)**	2.8	3.6	4.6	5.8	7.6	2.6	3.7	4.6	5.9	7.2	2.7	3.6	4.7	5.8	7.0	2.7	3.5	4.6	5.9	7.2
**MQ (kN/mm)**	6.5	4.4	3.0	2.1	1.4	6	4.3	2.9	2.2	1.5	5.8	4.1	2.9	2.1	1.5	6.1	4.2	3.1	2.2	1.4
**F_t_ (mm)**	1.0	1.8	2.5	3.3	3.9	1.1	1.8	2.6	3.2	3.6	1.1	1.9	2.5	3.1	3.5	1	1.9	2.6	3.2	3.7
**F_T_ (mm)**	3.0	4.1	4.8	6.6	8.0	2.9	4.2	4.9	6.5	7.6	2.8	4.1	5.0	6.3	7.4	2.8	4.2	4.9	6.7	7.7
**E (MPa)**	103.2	70.1	48.2	32.9	22.6	96.1	68.5	46.3	34.9	23.8	91.6	64.6	46.1	33.2	23.7	97.8	67.2	49.6	35.4	22.5
**maph (g)**	1226.0	1244.0	1256.0	1255.7	1259.4	1238.1	1241	1232.3	1248.1	1229.4	1223.4	1242.9	1256.3	1253.0	1253.8	1242.6	1229.5	1250.3	1234.7	1240.4
**Gagg (g/cm^3^)**	2.755	2.755	2.755	2.755	2.755	2.752	2.752	2.752	2.752	2.752	2.757	2.757	2.757	2.757	2.757	2.760	2.760	2.760	2.760	2.760
**Gmb (g/cm^3^)**	2.408	2.430	2.442	2.448	2.446	2.423	2.430	2.411	2.442	2.407	2.409	2.431	2.450	2.452	2.438	2.432	2.41	2.446	2.418	2.432
**Gmm (g/cm^3^)**	2.579	2.558	2.538	2.518	2.499	2.571	2.568	2.531	2.524	2.511	2.581	2.561	2.540	2.521	2.501	2.554	2.562	2.528	2.538	2.518
**V_a_ (%)**	6.6	5.0	3.8	2.8	2.1	6.5	5.2	3.7	2.8	2.4	6.7	5.1	3.6	2.7	2.5	6.6	5.1	3.7	2.9	2.3
**VFA (%)**	58.8	68.2	76.0	82.6	87.1	59	68.4	76.8	83.4	86.3	58.7	68.0	77.2	83.0	85.2	59.1	68	76.5	82.9	86.6
**VMA (%)**	16.1	15.8	15.8	16.0	16.5	16.2	15.7	15.8	16.2	16.7	16.1	15.8	15.6	16.0	16.9	16	15.6	15.7	16.2	16.6

## Data Availability

The original contributions presented in this study are included in the article/Supplementary Materials. Further inquiries can be directed to the corresponding author.

## Supplementary Materials

The following supporting information can be downloaded at: https://www.mdpi.com/article/10.3390/ma19143054/s1, Figure S1: SEM micrographs with EDS spectra of inclusions within the WETM sample; Figure S2: SEM micrographs with EDS spectra of inclusions within the WEART sample.
