# Supplementary material for: Innovative Bitumen Modification Technology Using Industrial Waste Enamels in Asphalt Mixtures Production
Source: Materials (Basel). 2026 Jul 15;19(14):3054. doi: 10.3390/ma19143054 (PMC13413565; doi:10.3390/ma19143054)
Supplement: Supplementary file 1 [file materials-19-03054-s001.zip › materials-4277109-supplementary.pdf]

Supplementary Materials_S1

Innovative bitumen modification technology using industrial waste enamels in processes production of asphalt mixtures

Miodrag Ristović[1], Jelena Gulicovski[1], Milan Kragović[1], Nenad Ristić[2], Ivica Ristović[3], Sanja Živković[1], Marija Stojmenović [1,*]

[1] Institute of Nuclear Sciences "Vinča"-National Institute of the Republic of Serbia, University of Belgrade, 22-24 Mike Petrovića Alasa, 11351 Belgrade, Serbia;
[2] Faculty of Civil Engineering and Architecture, University of Niš, Đušina 7, 18106 Niš, Serbia;
[3] Faculty of Mining and Geology, University of Belgrade, Aleksandra Medvedeva 14, 11000 Belgrade, Serbia;
* Correspondence: mpusevac@vinca.rs

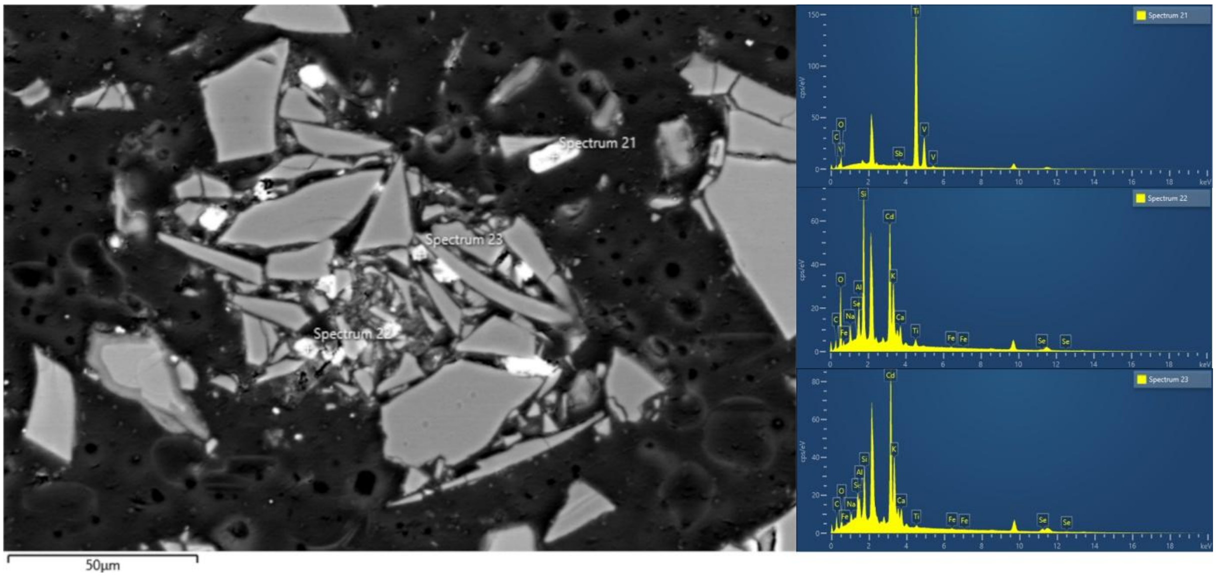

Figure S1. SEM micrographs with EDS spectra of inclusions within the WETM sample

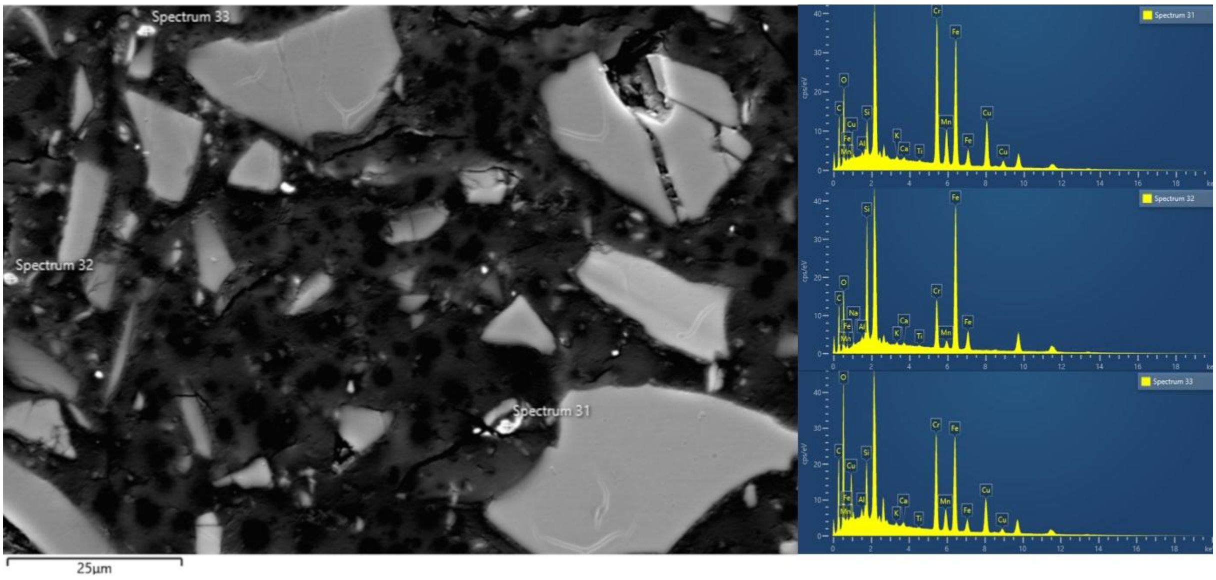

Figure S2. SEM micrographs with EDS spectra of inclusions within the WEART sample
